# Autoimmune cytopenias in chronic lymphocytic leukemia

**DOI:** 10.12669/pjms.35.5.369

**Published:** 2019

**Authors:** M. Shabih Haider, Saleem Ahmed Khan, Nasiruddin, Samra Shahid

**Affiliations:** 1 Dr. Mohammad Shabih Haider, MBBS, Department of Pathology (Haematology Section), Army Medical College, Rawalpindi, Pakistan; 2 Dr. Saleem Ahmed Khan, FCPS, Department of Pathology (Haematology Section), Army Medical College, Rawalpindi, Pakistan; 3 Dr. Nasiruddin, FCPS, Department of Pathology (Haematology Section), Army Medical College, Rawalpindi, Pakistan; 4 Dr. Samra Shahid FCPS, Department of Pathology (Haematology Section), Army Medical College, Rawalpindi, Pakistan

**Keywords:** Autoimmune granulocytopenia, Autoimmune hemolytic anaemia, Chronic lymphocytic leukemia, Immune thrombocytopenic purpura, Pure red cell aplasia

## Abstract

**Objective:**

To determine the frequency of autoimmune cytopenias in chronic lymphocytic leukemia.

**Methods:**

This cross sectional study was carried out at Department of Hematology, Army Medical College Rawalpindi, in collaboration with Military Hospital Rawalpindi and Armed Forces institute of Pathology Rawalpindi from 1st January 2018 to 1st October 2018. Sample size of 64 was calculated using WHO calculator. Age and gender of patients was noted. Frequency of autoimmune hemolytic anaemia, immune thrombocytopenic purpura, pure red cell aplasia and autoimmune agranulocytosis were determined in diagnosed patients of chronic lymphocytic leukemia by various laboratory tests in our study population.

**Results:**

A total of 64 patients were included in the study, 53 (82.8%) were males and 11(17.2%) were females. Mean age of patients was 65 years. Autoimmune hemolytic anaemia was observed in 5/64 (7.8%) of patients. Immune thrombocytopenic purpura was seen in 2/64 (3.1%) patients. Autoimmune granuloytopenia and pure red cell aplasia were not seen in any patient.

**Conclusion:**

Autoimmune hemolytic anaemia and immune thrombocytopenic purpura are the most common causes of immune cytopenias in patients of CLL. Immune cytopenias should always be identified by laboratory tests as their management differs from other cytopenias which occur due to various other causes.

## INTRODUCTION

Chronic lymphocytic leukemia is a significant sub group of chronic leukemias in adults.^1^ Chronic lymphocytic leukemia patients present with lymphadenopathy and peripheral blood lymphocytosis > 5 x 10^9^/L which is characterized by CD 5+, CD19+ and CD23+ on immunophenotyping.^2^ These lymphocytes are morphologically mature but functionally incompetent. The disease has a peak incidence between 60 to 80 years of age. It is more prevalent in the western part of the world where it predominates in males.^3^

Pathogenesis of chronic lymphocytic leukemia involves impaired apoptosis and prolonged survival of lymphocytes. There is accumulation of mature looking lymphocytes in blood, bone marrow, liver, spleen and lymph nodes. Monoclonal B lymphocytosis is an essentially pre-leukemic state of CLL which represents asymptomatic proliferation of clonal B cells with circulating numbers < 5x10^9^/L and absence of lymphadenopathy or disease-related symptoms. The risk of monoclonal B lymphocytosis changing to chronic lymphocytic leukemia is affected by polymorphism of genes controlling key functions in B cell development.^4^

Some of the chronic lymphocytic leukemia patients can present with cytopenias. These cytopenias include autoimmune hemolytic anaemia, immune thrombocytopenic purpura, pure red cell aplasia and autoimmune agranulocytosis.^5^ Amongst CLL associated cytopenias autoimmune hemolytic anaemia is the most common.^6^ Autoimmune hemolytic anaemia is characterized by production of antibody against patient’s own red cells. It is characterized by a positive direct antiglobulin test also known as Direct Coombs test along with raised serum bilirubin, increased reticulocytes and presence of spherocytes on examination of the peripheral blood film.^7^

Immune thrombocytopenic purpura is second most common presentation of cytopenia in chronic lymphocytic leukemia. It is characterized by thrombocytopenia with platelet count of < 100 x 10^9^/L in the peripheral blood. The bone marrow shows normal or increased number of megakaryocytes, in the absence of splenomegaly, infection or chemotherapy during previous one month.^8^ Anti platelet antibody test is not routinely used for diagnosis of chronic lymphocytic leukemia associated immune thrombocytopenic purpura because it is expensive and non-specific and not available in many diagnostic laboritories.^9^ Pure red cell aplasia and autoimmune granulocytopenia are extremely rare complications of chronic lymphocytic leukemia.^4^

Various studies in the west have reported frequencies of cytopenias but there are very few studies in our part of the world denoting these frequencies. The objective of this study was to determine the frequency of autoimmune cytopenias by standard laboratory investigations in diagnosed patients of chronic lymphocytic leukemia in our setup.

This study will help in distinguishing autoimmune cytopenias from cytopenias due to bone morrow infiltration as management of both types of cytopenias is fundamentally different according to international workshop on CLL 2008 guidelines.^2^ These guidelines recommended that CLL patients with autoimmune cytopenias should be treated with glucocorticoids. Second line treatment options include splenectomy, intravenous steroids and high dose immunosuppressive agents such as cyclosporine.

## METHODS

This cross sectional study was conducted at department of Haematology, Army Medical College in collaboration with Military Hospital Rawalpindi and AFIP Rawalpindi from 1st January 2018 to 1st October 2018. Permission from the Institutional review board and ethical committee were obtained. Confidence interval was kept at 95%, anticipated population as 4.5%, absolute precision required as 5%. Sample size of 64 was calculated by WHO calculator. Sampling technique was non probability purposive sampling.

Patients of Chronic lymphocytic leukemia of both genders without previously known autoimmune diseases or recent transfusion reactions were included in the study irrespective of age, marital status and stage of the disease. All the patients with previously known autoimmune diseases such as Rheumatoid arthritis, Systemic lupus erythematous, Graves’ disease, Hashimoto’s thyroiditis, Type-1 Diabetes Mellitus were excluded from the study.

Informed consent was taken. Demographic features of the patients were noted. Stage of disease according to Rai classification was noted. Nine (9) ml of venous blood was drawn under aseptic conditions. Five (5) ml were transferred to EDTA tube for complete blood counts, peripheral smear examination and direct Coombs test. Four (4) ml were transferred to plain tube/ clot activator for serum analysis. Serum was separated by centrifugation at 4000 rpm for five minutes and stored at -20°C until biochemical analysis.

Complete blood counts were generated by Sysmex KX-21 TM Automated haematology analyzer with adequate quality control. Peripheral blood smears were examined after staining with leishman stain. Reticulocyte were counted and expressed as percentage after supravital staining by Brilliant cresyl blue stain. Direct coombs test was carried out using Anti human globulin reagent (Coombs Reagent).

Serum Bilirubin and LDH levels were measured by Selectra XL. Bone marrow examination was done to investigate the cytopenias, slides were stained with leishman stain. Anti nuclear antibody test, rheumatoid factor antibody test and anti neutrophil cytoplasmic antibody test was done by indirect immunofluorescence method. Thyroid function tests and thyroid peroxidase antibody test was done to exclude Hashimoto thyroiditis. Glycosylated haemoglobin test was done to rule out Type-1 Diabetes Mellitus.

Based on these laboratory tests, autoimmune hemolytic anaemia was diagnosed as positive coombs test and any two other signs of hemolysis including increased LDH, reticulocytes and bilirubin^7^. Immune thrombocytopenic purpura was diagnosed as a diagnosis of exclusion. It was diagnosed as presence of thrombocytopenia with presence of normal or increased number of megakaryocytes in bone marrow without previous chemotherapy, splenomegaly or other autoimmune disease.^8^ Red cell aplasia was diagnosed as isolated absolute reticulocytopenia with complete absence of erythroid precursors in bone marrow.^7^ However, in CLL reticulocytopenia and depression in erythroid series also occurs due to bone marrow infiltration. Hence in all those case in which there was bone marrow infiltration, possibility of red cell aplasia was not considered. Autoimmune granulocytopenia was diagnosed as absence of myeloid precursors in bone marrow, with positive anti neutrophil cytoplasmic antibodies and exclusion of other autoimmune diseases.^5^

### Statistical Analysis

Data was analyzed by statistical package for social sciences (SPSS 23). For qualitative variables frequency and percentages were calculated and for quantitative variables mean and standard deviation were calculated.

## RESULTS

**Fig.1:**
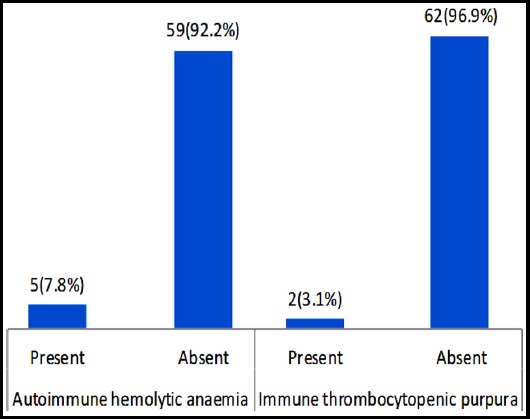
Frequency of autoimmune hemolytic anaemia and immune thrombocytopenic purpura (n=64).

A total of 64 patients were included in our study. The mean age of patients was 65 years. Out of 64 patients, 53 (82.8%) were male and 11(17.2%) were female. Autoimmune hemolytic anaemia was seen in 5/64 (7.8%) of patients. Immune thrombocytopenic purpura was seen in 2/64 (3.1%) patients (Fig.1). Autoimmune granulocytopenia and pure red cell aplasia were not seen in any of our patients. Based upon Rai system of classification 8 (13%) patients were in Stage 1, 7 (11%) patients in Stage 2, 20 (31%) patients in Stage 3 and 29 (45%) patients in stage 4.

Mean Hemoglobin was 10.65±2.66 g/dl (range 3.60-16.0). Mean total leucocyte count was 88.743 ±106.88 x 10^9^/l (range 11-690). Mean platelet count was 142.457 ±73.03 x10^9^/l (range 8.00-383). Mean reticulocyte count was 1.79±1.25 (range 0.20-6.0). Mean serum LDH was 298 ± 135.08 U/l (range 50-698). Mean Absolute Lymphocytic count was 77.01± 94.91x10^9^/l (range 8.19-566). Mean total bilirubin was 16.5±35.88 umol/L (range 4.0 to 285). Mean Indirect Bilirubin was 8.18±11.85 umol/L (range 2- 90). Mean direct Bilirubin was 8.29 umol/L ± 24.60 (range 2.00 to 195.0) (Table-I). Coombs test was negative in 56 (87.5%) of patients. In 4 (6.3%) patient it was 1+ positive, in 3 patients (4.7%) it was 2+ positive, in 1 (1.6%) patient it was 3+ positive.

**Table I:** Mean and standard deviation of quantitative variables (n=64).

	Minimum	Maximum	Mean	Std. Deviation
Age (in years)	50.00	87.00	66.54	± 9.47
Haemoglobin(g/dl)	3.60	16.00	10.64	± 2.66
TLC (x 10^9^/L)	11.00	690.90	88.74	± 106.87
Platelets(x 10^9^/L)	8.00	383.00	142.45	± 73.031
Retics	0.20	6.00	1.80	± 1.25
LDH(U/L)	150.00	698.00	298.11	± 135.09
Absolute lymphocyte count(x 10^9^/L)	8.19	566.00	77.01	± 94.91
Total Bilirubin(umol/L)	4.00	285.00	16.50	± 35.88
Indirect Bilirubin(umol/L)	2.00	90.00	8.19	± 11.85
Direct Bilirubin(umol/L)	2.00	195.00	8.30	±24.61

## DISCUSSION

Diagnosis of chronic lymphocytic leukemia requires > 5x10^9^/L lymphocytes in peripheral blood. The immunophenotype of B lymphocytes is defined by flow cytometry as CD5+, CD 19+ and CD 23+. The cells in chronic lymphocytic leukemia are small mature lymphocytes with narrow border of cytoplasm.^10^

The patients of chronic lymphocytic leukemia can present with autoimmune cytopenias.^10^ Pathophysiological mechanisms involved in the development of autoimmune cytopenias in chronic lymphocytic leukemia include loss of self-tolerance and aberrant T- and B-cell function, resulting in auto antigen presentation by malignant CLL cells, antibody production by normal B cells and reduced immunity through loss of regulatory T-cells.^11^

Our results show that frequency of autoimmune cytopenias is comparable to that reported in the international studies.^12^ As for autoimmune hemolytic anaemia our frequency of 7.8% is comparable to a study by Moreno C et al. which shows frequency of autoimmune hemolytic anaemia as 7%.^13^ Our patients who developed autoimmune hemolytic anaemia were mostly male 80% (4/5). Both patients who developed Immune thrombocytopenic purpura were also male. All the patients who developed these autoimmune complications were above 50 years of age and in stage 3 or stage 4. Hence our results are comparable to international data which shows association of CLL with male sex, high lymphocytic count and advanced disease.^13^,^14^

As for immune thrombocytopenic purpura our frequency result of 3.1% is slightly lower than a study conducted by Visco C et al., in which 5% of patients of CLL during the course of disease developed ITP.^8^ On the other side, our study is comparable with a study conducted by Ehsan A et al. which shows frequency of ITP as 3.2%.^15^ However, in that study only 31 patients of CLL were included in the study.

Autoimmune granulocytopenia and pure red cell aplasia have rarely been reported in chronic lymphocytic leukemia. In a study conducted by Zent CS et al. < 0.5% of patients presented with pure red cell aplasia or autoimmune granulocytopenia.^16^ Similarly, in a study conducted by Monero C et al. none of the patients developed autoimmune granuloytopenia or pure red cell aplasia.^13^

Dearden C et al., studied the prognostic effect of a positive Coombs test (DAT) in patients with CLL.^17^ A positive Coombs test (DAT) predicted a poorer response to treatment and together with AIHA was associated with a lower overall survival. Regarding ITP, Visco C et al. reported that this complication was associated with an inferior outcome in CLL patients.^8^

Rai and Binet systems of classification in staging CLL take into account the cytopenias however they do not provide information regarding the cause of cytopenias. Our study helped in distinguishing autoimmune cytopenias from cytopenias due to bone marrow infiltration as management of both these type of cytopenias is fundamentally different.^18^

### Limitations of study

Limitation of our study is that it was carried out on a small sample size. However, it is still recommended that all patients of CLL should undergo autoimmune investigations in all those centres involved in management of CLL. After a large cohort of patients is studied and meta-analysis of results is done, it may help in exploration of results to whole Pakistani population.

## CONCLUSION

Autoimmune hemolytic anaemia and immune thrombocytopenic purpura are the most common causes of immune cytopenias in chronic lymphocytic leukemia. They should always be investigated by laboratory tests as their management is different from other complications that occur due to bone marrow infiltration in CLL.

### Author`s Contribution

**MSH** did data collection, statistical analysis and manuscript writing.

**SAK, N and SS** did review and final approval of manuscript.
